# Physical Activity and Sedentary Time in Active and Non-Active Adults with Intellectual Disability: A Comparative Study

**DOI:** 10.3390/ijerph16101761

**Published:** 2019-05-18

**Authors:** Guillermo R. Oviedo, Nauris Tamulevicius, Myriam Guerra-Balic

**Affiliations:** 1 Faculty of Psychology, Education and Sport Science-Blanquerna, University Ramon Llull, 34 Císter Street, 08022 Barcelona, Spain; miriamelisagb@blanquerna.url.edu; 2 Faculty of Health Sciences Blanquerna, University Ramon Llull, 326-332 Padilla Street, 08025 Barcelona, Spain; 3 Department of Health Sciences and Human Performance, College of Natural and Health Sciences, The University of Tampa, Tampa, FL 33606, USA; ntamulevicius@ut.edu

**Keywords:** intellectual disability, adults, physical activity, sedentary lifestyle, accelerometer

## Abstract

This study describes and compares physical activity (PA) levels and sedentary time (ST) of active (AG) and a non-active (NAG) groups of adults with intellectual disability (ID) versus a group of adults without ID. Thirty-seven participants from the AG, 29 from the NAG, and 31 adults without ID participated in this study. Height and weight were obtained to calculate body mass index (BMI). PA levels and ST were assessed with GT3X Actigraph accelerometers for 7 days. Results revealed that the AG engaged in higher values of moderate to vigorous PA compared with the NAG (all *p* < 0.05), but were similar to adults without ID. Adults without ID performed less ST and more light PA than the ID groups (all *p* < 0.05). The participants of the AG did not demonstrate less ST than the NAG. It is concerning that adults with ID (AG or NAG) are spending a higher time in ST and less time in light PA than adults without ID. Our results suggest that integrated, well-designed PA programmes into the ID population workdays can lead to increased PA levels. Nevertheless, these interventions and exercise programmes implemented for adults with ID should be tailored to also reduce ST.

## 1. Introduction

The benefits of physical activity (PA) on health have been well documented and different health guidelines have been published suggesting that people should perform a certain amount of PA in order to obtain health-related benefits [1,2]. There has also been an increased interest in evaluating PA and its nature in different populations [1,3].

There are currently many measurement devices such as pedometers, accelerometers, heart-rate monitors, and inclinometers that provide detailed information about intensity, frequency, and duration of PA [4,5]. This has opened up a new domain of PA research that focuses on the effects of different PA intensities and duration as well as the distribution of varying intensities of PA across certain time frames (day, week, year) [6].

Evidence is accumulating that the maintenance of health must not simply be deduced from the total amount of activity spent in moderate to vigorous PA (MVPA) but also in the amount of time spent in sedentary or light-intensity PA [7]. The Sedentary Behaviour Research Network has defined sedentary behaviour as activities with energy expenditures of ≤1.5 metabolic equivalents (MET) while in a sitting, reclining, or lying posture during waking hours [8]. Sedentary behaviour has been increasingly recognized as an independent risk factor for premature death and numerous chronic health conditions [9]. Although possibly not as beneficial as MVPA, substituting sedentary time (ST) with light PA (LPA) has also been shown to independently reduce mortality [10].

Adults with intellectual disabilities (ID) have a high prevalence of non-communicable diseases and earlier mortality when compared with the general population [11,12,13,14,15]. They are also at further increased risk due to having lower levels of PA, decreased cardiovascular fitness, and poorer nutritional habits [16,17,18,19]. Individuals with ID also experience different personal and environmental barriers that preclude them from doing PA and reducing ST [20,21,22]. Previous studies have shown that people with ID get old earlier than the general population and show signs of aging in their 40 s and 50 s [23,24]. In Spain, adults with ID older than 40–45 years old are considered older adults because the frequency of suffering physical health problems, musculoskeletal disability, visual, and hearing problems are similar to people older than 65 years old without ID [25].

In adults with ID, the total amount of PA has been demonstrated to be very low. The study by Dairo et al. [17] found that 9% of adults with ID met the World Health Organization (WHO) recommendations on PA for health, while 7–45% took ≤10,000 steps per day with an average of 6851 steps per day. Furthermore, another review by Melville et al. [26] found that adults with ID spent between 522 and 643 min per day in ST.

As far as we know, detailed information on PA levels and ST in active and non-active adults with ID in comparison with adults without ID is sparse, despite the relevance of the knowledge in terms of planning effective interventions and evaluation of PA programmes for this target group. Furthermore, no studies have compared if PA programmes implemented at centres for adults with ID are increasing their daily PA levels and reducing their ST in comparison with adults without disabilities.

Therefore, the main objective of this study was to describe and compare the PA levels and ST of active adults with ID (i.e., participating in PA programmes), non-active adults with ID (i.e., not participating in PA programmes), and adults without ID. A secondary purpose was to analyse the potential age and sex differences in PA levels and ST among groups. Finally, an objective of this study was to determine variations in time spent in different intensities of PA and ST throughout the week and compare them across the groups.

## 2. Materials and Methods

### 2.1. Study Design and Participants

This study is a cross-sectional design comparing active and non-active adults with ID with a group of adults without ID. A total of 92 adults (51 male/41 female), between 20 to 60 years old from occupational day centres, with mild to moderate ID and 50 adults without ID (25 male/25 female) were invited to participate in this study. All participants were able to walk without aids and did not present motor impairments. To be included in this study, the participants from the active groups (AG) should be part of the PA programmes offered by their centres; the participants from the non-active groups (NAG) should not be participating in exercise programmes nor performing other PA activities outside their centres, and the adults without ID should not be part of exercise programmes, nor belong to sport teams. Exclusion criteria included contraindications to exercise and medication that may have an important effect on their activities of daily living. Daily living activities at the centres, from 9:00 am until 5:00 pm with a break of 1 h for lunch, included activities such as sewing rugs, arts and crafts, weaving baskets, computer work, watching TV or videos, cleaning, and gardening.

The ID classification was obtained from the patients’ medical records, and all the participants were diagnosed with mild to moderate ID according to the Spanish National Government classification [27]. This classification of ID represents a combination of patients’ level of intelligence quotient (IQ) and adaptive behaviour, and categorizes the percentage of disability (physical, intellectual and/or sensorial) in five degrees as follows: non-existent (0%), borderline (15–29%), mild (30–59%), moderate (60–75%), and severe or very severe (≥76%).

Informed consent to participate in this study were obtained from 27 females and 39 males with ID (43 ± 12 years old) from their parents/legal guardians, and from 17 females and 14 males without ID (43 ± 11 years old).

The group of adults without ID consisted of 31 participants. All of them did not present disabilities, and were not part of PA programmes nor sport teams.

In the active group (AG) 37 participants were included. At their centres, the participants from the AG took part in PA programmes three times a week (Monday, Wednesday, and Friday), 1 h per session.

The non-active group (NAG) consisted of 29 participants diagnosed with ID who did not participate in exercise programmes.

### 2.2. Procedures

Two meetings were held with the participants and parents/legal guardians. During the first meeting, objectives, procedures, and length of the study were explained to the participants and parents/legal guardians. Prior to participation, all participants and parents and/or legal guardians who wanted to be included in this study signed an informed consent form. During the second meeting, the anthropometric assessments were carried out and the accelerometers were fitted on the right hip of the participants.

This study was approved by the Institutional Research Ethics Committee (Ethical code: CER_URL_2017_2018_005) and follows the Helsinki guidelines for ethical behaviour.

### 2.3. Anthropometric Measurements

Height was measured to the nearest 0.1 cm using a stadiometer (Seca 225, Seca, Hamburg, Germany). Weight was measured to the nearest 0.1 kg on a digital scale (Seca 861, Hamburg, Germany) with the subject wearing lightweight clothing and no shoes. Body mass index (BMI) was calculated as weight in kilograms divided by height in meters squared (kg/m^2^).

### 2.4. Physical Activity Level Measurements

Physical activity was assessed by using the GT3X ActiGraph accelerometer (Firmware 4.4.0, ActiGraph, Fort Walton Beach, FL, USA) and data were downloaded with the ActiLife 6 Software v.6.12.0. The devices were programmed to measure activity in 5 s (5-s epochs) and successively converted into a 60-s epoch. Outcome variables were total PA (TPA) (counts·min^−1^), time spent (min·day^−1^) in ST (0 to 99 counts·min^−1^), LPA (100 to 2019 counts·min^−1^), moderate PA (MPA) (2020 to 5998 counts·min^−1^), and vigorous PA (VPA) (≥5999 counts·min^−1^). Time in MVPA was determined by adding MPA to VPA. Additionally, accelerometer data were divided into the seven days of the week. The intensity cut-off points were replicated from the National Health and Nutritional Examination Survey (NHANES) for adults over 18 years of age [28].

The proportion of participants meeting World Health Organization (WHO) global recommendations on PA for health [2], which specify that adults should do at least 150 min of moderate intensity aerobic PA or 75 min of vigorous intensity aerobic PA throughout the week or an equivalent combination of moderate and vigorous intensity activity in bouts of at least 10 min, was assessed. 

To be considered as having valid data, participants must have worn the accelerometer during waking hours for ≥10 h/day [29,30] and for ≥4 (week and/or weekend) days [31,32]. Non-wearing time was defined as a period of 60 consecutive minutes of zero counts with an allowance for up to 2 min of counts between 1 and 100.

All participants and parents/legal guardians received face to face instructions on how to wear the accelerometer (correct positioning and orientation of the accelerometer) during all waking hours and wear time. Instructions were reinforced with an information sheet given to each participant and parents/legal guardians. The accelerometers were preprogrammed to collect data during the 7 days. The devices were fitted to the participants’ right hip and were collected 8 days later. The devices were not used while bathing or swimming.

### 2.5. Statistical Analysis

Descriptive statistics were calculated for all variables. To test the normality of the variables, the Kolmogorov–Smirnov test was utilized. A chi-square test of independence was performed to examine the relation between groups and ID levels. Variables of age and anthropometry were analysed by using a one-way analysis of variance (ANOVA).

Total PA, PA levels, and ST of each group were compared by using a one-way analysis of covariance (ANCOVA). Secondly, based on previous literature [23,24] all three groups were subdivided by age (<40 years old vs. ≥40 years old) and sex, and differences were analysed by using a one-way ANCOVA.

Finally, a linear mixed-effects model was performed to analyze interactions between PA levels, ST, and groups throughout the week. Since we used a linear mixed-effects model, we did not have to exclude from the analysis the few individuals for whom the outcome variable was not available at one time point. Post-hoc pairwise comparisons were employed to analyse between-group differences.

All models were controlled for accelerometer wearing time and effect size (Cohen’s *d*) was calculated when possible.

The critical values for statistical significance were assumed at an alpha level ≤0.05. Statistical analyses were conducted using the Statistical Package for the Social Sciences (SPSS) v22.0 (IBM SPSS Statistics, Chicago, IL, USA).

## 3. Results

### 3.1. Participants Characteristics, Anthropometrics, and PA Levels

Out of the 97 participants, 2 participants from the AG and 1 participant from the NAG dropped out and therefore the final sample was made up of 37 participants from the AG, 29 from the NAG, and 31 from the group of adults without ID. The level of ID was equally distributed over the two groups with ID (*X*^2^ = 0.256; *p* = 0.613) (Table 1).

The general characteristics of the participants are presented in Table 2. The group of adults without ID was taller than the rest of groups (non-ID group vs. AG: *t* = 2.084, *p* = 0.040, *d* = 0.78; non-ID group vs. NAG: *t* = 2.810, *p* = 0.006, *d* = 0.69).

Table 2 also presents the characteristics and the mean values of PA data collected during a week for all the three groups. The AG and the adults without ID performed more TPA than the NAG (AG vs. NAG: *t* = 3.055, *p* = 0.003, *d* = 0.73; non-ID group vs. NAG: *t* = 3.393, *p* = 0.001, *d* = 0.86). Adults without ID spent less time in ST (non-ID group vs. AG: *t* = 4.481, *p* < 0.001, *d* = 1.06; non-ID group vs. NAG: *t* = 4.241, *p* < 0.001, *d* = 1.25) and more time in LPA than the groups of adults with ID (non-ID group vs. AG: *t* = 10.114, *p* < 0.001, *d* = 2.42; non-ID group vs. NAG: *t* = 8.198, *p* < 0.001, *d* = 1.89). The AG engaged in significantly higher amounts of MPA (*t* = 2.212, *p* = 0.021, *d* = 0.52) and MVPA (*t* = 2.282, *p* = 0.018, *d* = 0.53) than the NAG. In addition, the AG performed more VPA than the NAG (*t* = 2.164, *p* = 0.033, *d* = 0.71) and the group of adults without ID (*t* = 2.174, *p* = 0.032, *d* = 0.53). The number of sedentary bouts per day was greater in all groups of adults with ID than participants without ID (non-ID group vs. AG: *t* = 3.142, *p* = 0.002, *d* = 1.08; non-ID group vs. NAG: *t* = 3.164, *p* = 0.002, *d* = 1.12).

### 3.2. Physical Activity and Sedentary Time Differences by Sex

Sedentary time and PA outcomes of each group by sex are shown in Table 3. Males from the active and from the non-ID group engaged in more total PA than males (AG vs. NAG: *t* = 2.315, *p* = 0.025, *d* = 0.63; non-ID group vs. NAG: *t* = 2.410, *p* = 0.019, *d* = 0.75) and females (AG vs. NAG: *t* = 2.678, *p* = 0.009, *d* = 1.02; non-ID group vs. NAG: *t* = 2.758, *p* = 0.007, *d* = 1.38) from the NAG. Females without ID performed higher amounts of total PA than females from the NAG (*t* = 2.448, *p* = 0.018, *d* = 1.38). Males and females from the group of adults without ID spent less waking time in ST (males without ID vs. males from the AG: *t* = 3.602, *p* < 0.001, *d* = 1.33; males without ID vs. females from the AG: *t* = 2.919, *p* < 0.001, *d* = 1.09; males without ID vs. males from the NAG: *t* = 2.848, *p* < 0.001, *d* = 1.27; males without ID vs. females from the NAG: *t* = 3.561, *p* < 0.001, *d* = 2.12; females without ID vs. males from the AG: *t* = 3.300, *p* < 0.001, *d* = 1.00; females without ID vs. females from the AG: *t* = 2.596, *p* < 0.001, *d* = 0.80; females without ID vs. males from the NAG: *t* = 2.512, *p* < 0.001, *d* = 0.86; females without ID vs. females from the NAG: *t* = 3.279, *p* < 0.001, *d* = 1.33) and engaged in more LPA (males without ID vs. males from the AG: *t* = 6.046, *p* < 0.001, *d* = 2.00; males without ID vs. females from the AG: *t* = 6.027, *p* < 0.001, *d* = 2.45; males without ID vs. males from the NAG: *t* = 4.798, *p* < 0.001, *d* = 1.49; males without ID vs. females from the NAG: *t* = 4.761, *p* < 0.001, *d* = 1.86; females without ID vs. males from the AG: *t* = 8.282, *p* < 0.001, *d* = 2.48; females without ID vs. females from the AG: *t* = 8.019, *p* < 0.001, *d* = 2.89; females without ID vs. males from the NAG: *t* = 6.845, *p* < 0.001, *d* = 1.97; females without ID vs. females from the NAG: *t* = 6.549, *p* < 0.001, *d* = 2.32) than males and females from the ID groups. Males from the AG performed more MPA and MVPA than males and females from the NAG (MPA: males from AG vs. males from the NAG, *t* = 2.656, *p* = 0.013, *d* = 0.68; males from the AG vs. females from the NAG, *t* = 2.157, *p* = 0.026, *d* = 0.66. MVPA: males from AG vs. males from the NAG, *t* = 2.715, *p* = 0.011, *d* = 0.78; males from the AG vs. females from the NAG, *t* = 2.228, *p* = 0.021, *d* = 0.81). Males and females from the AG and males from the adults without ID group performed more VPA than females from the NAG group (males from the AG vs. females from the NAG, *t* = 2.227, *p* = 0.028, *d* = 0.75; females from the AG vs. females from the NAG, *t* =1.313, *p* = 0.045, *d* = 0.51; males without ID vs. females from the NAG, *t* = 1.550, *p* = 0.042, *d* = 0.34). Only males from the AG performed more VPA than males from the NAG (*t* = 2.715, *p* = 0.032, *d* = 0.83). The number of bouts in sedentary behaviours per day was greater in males and females from all groups with ID than the group without ID (males from the AG vs. males without ID, *t* = 2.437, *p* = 0.017, *d* = 1.22; males from the AG vs. females without ID, *t* = 2.400, *p* = 0.018, *d* = 1.03; females from the AG vs. males without ID, *t* = 2.058, *p* = 0.043, *d* = 1.06; females from the AG vs. females without ID, *t* = 2.044, *p* = 0.044, *d* = 0.89; males from the NAG vs. males without ID, *t* = 2.470, *p* = 0.015, *d* = 1.14; males from the NAG vs. females without ID, *t* = 2.481, *p* = 0.014, *d* = 0.99; females from the NAG vs. males without ID, *t* = 2.023, *p* = 0.046, *d* = 1.19; females from the NAG vs. females without ID, *t* = 2.002, *p* = 0.048, *d* = 0.97).

### 3.3. Physical Activity and Sedentary Time Differences by Age

Table 4 depicts PA and ST outcomes from all the three groups divided by age. The youngest adults from the AG and from the non-ID group performed greater amounts of total PA than the youngest and oldest groups from the NAG (<40 years from the AG vs. <40 years from the NAG, *t* = 2.803, *p* = 0.005, *d* = 0.90; <40 years from the AG vs. ≥40 years from the NAG, *t* = 3.014, *p* = 0.003, *d* = 0.93; <40 years without ID vs. <40 years from the NAG, *t* = 2.856, *p* = 0.007, *d* = 1.00; <40 years without ID vs. ≥40 years from the NAG, *t* = 3.064, *p* = 0.003, *d* = 1.01). More total PA was performed by the oldest group of adults without ID than the oldest group from the NAG (*t* = 2.059, *p* = 0.046, *d* = 0.74). For ST and LPA, significant differences were only found between the youngest and oldest participants from the non-ID group when compared with both age groups from the AG and NAG (ST: <40 years without ID vs. <40 years from the AG, *t* = 2.375, *p* < 0.001, *d* = 1.08; <40 years without ID vs. ≥40 years from the AG, *t* = 5.241, *p* < 0.001, *d* = 1.60; <40 years without ID vs. <40 years from the NAG, *t* = 3.780, *p* < 0.001, *d* = 1.50; <40 years without ID vs. ≥40 years from the NAG, *t* = 3.333, *p* < 0.001, *d* = 1.39; ≥40 years without ID vs. <40 years from the AG, *t* = 1.397, *p* < 0.001, *d* = 0.75; ≥40 years without ID vs. ≥40 years from the AG, *t* = 4.325, *p* < 0.001, *d* = 1.20; ≥40 years without ID vs. <40 years from the NAG, *t* = 2.852, *p* < 0.001, *d* = 1.09; ≥40 years without ID vs. ≥40 years from the NAG, *t* = 2.462, *p* < 0.001, *d* = 0.99. LPA: <40 years without ID vs. <40 years from the AG, *t* = 8.371, *p* < 0.001, *d* = 3.12; <40 years without ID vs. ≥40 years from the AG, *t* = 7.543, *p* < 0.001, *d* = 2.84; <40 years without ID vs. <40 years from the NAG, *t* = 7.389, *p* < 0.001, *d* = 2.67; <40 years without ID vs. ≥40 years from the NAG, *t* = 5.575, *p* < 0.001, *d* = 2.01; ≥40 years without ID vs. <40 years from the AG, *t* = 6.904, *p* < 0.001, *d* = 2.13; ≥40 years without ID vs. ≥40 years from the AG, *t* = 6.080, *p* < 0.001, *d* = 1.89; ≥40 years without ID vs. <40 years from the NAG, *t* = 5.943, *p* < 0.001, *d* = 1.79; ≥40 years without ID vs. ≥40 years from the NAG, *t* = 4.202, *p* < 0.001, *d* = 1.27). The youngest participants of the AG performed greater amounts of VPA than participants from the NAG (<40 years from the AG vs. <40 years from the NAG, *t* = 2.174, *p* = 0.025, *d* = 1.05; <40 years from the AG vs. ≥40 years from the NAG, *t* = 2.251, *p* = 0.020, *d* = 1.13). Also, the youngest participants from the AG performed more VPA than oldest participants from non-ID group (*t* = 3.136, *p* = 0.002, *d* = 1.35).

Youngest and oldest participants from the ID groups had a greater number of sedentary bouts per day than participants from the group of adults without ID (<40 years from the AG vs. <40 years without ID, *t* = 2.832, *p* = 0.006, *d* = 1.14; ≥40 years from the AG vs. <40 years without ID, *t* = 4.450, *p* < 0.001, *d* = 2.14; <40 years from the NAG vs. <40 years without ID, *t* = 3.128, *p* = 0.003, *d* = 1.60; ≥40 years from the NAG vs. <40 years without ID, *t* = 3.952, *p* = 0.002, *d* = 1.60).

### 3.4. Physical Activity and Sedentary Time Throughout the Week

**Figure 1:**
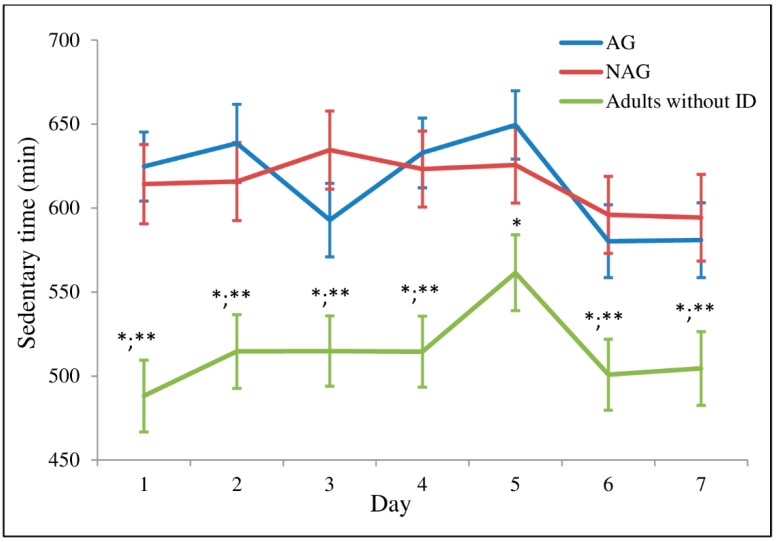
Time spent in sedentary time throughout the week. 1 (Monday); 2 (Tuesday); 3 (Wednesday); 4 (Thursday); 5 (Friday); 6 (Saturday); 7 (Sunday). Values are means (standard error); *n* = 35 for AG (active group); *n* = 28 for NAG (non-active group); *n* = 31 for adults without ID. * Significant difference (*p* ≤ 0.05) between adults without ID vs. AG; ** Significant difference (*p* ≤ 0.05) between adults without ID vs. NAG.

The analysis using linear mixed-effects models did not show a significant interaction between factors time and group for time spent in ST (time-by-group: *F* = 1.004, *p* = 0.444). Figure 1 shows the ST of all groups throughout the week. The group of adults without ID spent significantly lower time in ST on all days, except on Friday when the difference was only observed within the AG. No difference was found between the AG and NAG.

**Figure 2:**
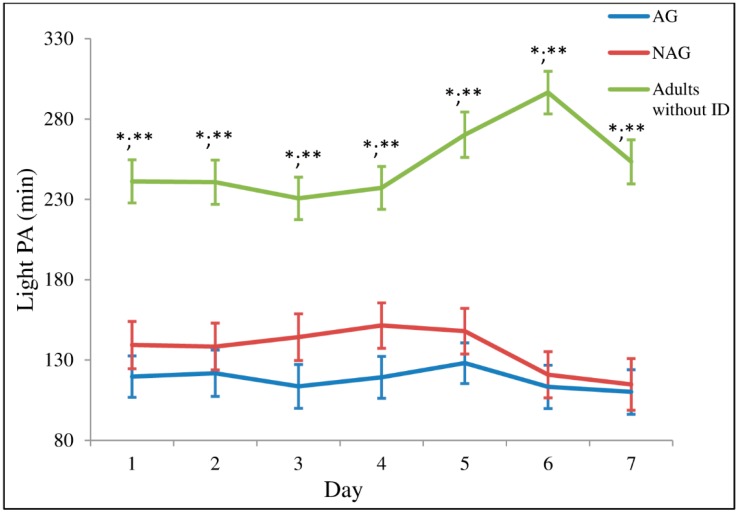
Time spent in light physical activity throughout the week. 1 (Monday); 2 (Tuesday); 3 (Wednesday); 4 (Thursday); 5 (Friday); 6 (Saturday); 7 (Sunday). Values are means (standard error); *n* = 35 for AG (active group); *n* = 28 for NAG (non-active group); *n* = 31 for adults without ID. * Significant difference (*p* ≤ 0.05) between adults without ID vs. AG; ** Significant difference (*p* ≤ 0.05) between adults without ID vs. NAG.

The analysis using linear mixed-effects models showed a significant interaction between factor time and group for LPA (time-by-group: *F* = 2.764, *p* = 0.001). Figure 2 shows the LPA performed by all groups. In general, the group of adults without ID performed more LPA than the rest of the groups throughout the week. No differences were observed between the AG and NAG.

**Figure 3:**
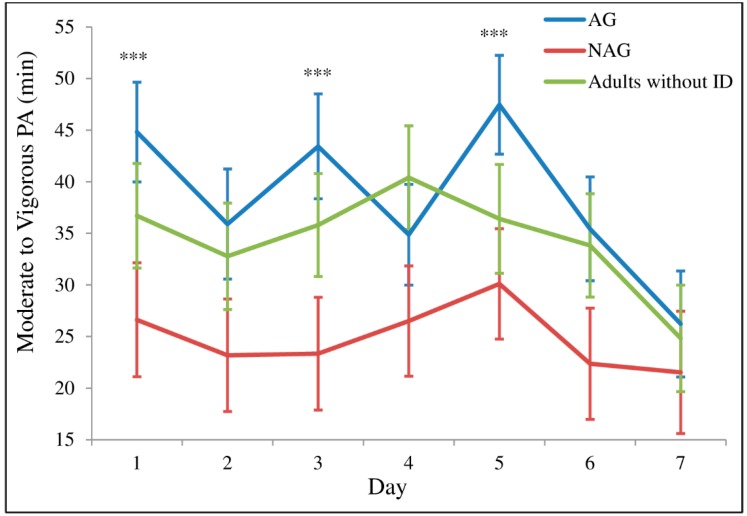
Time spent in moderate to vigorous physical activity throughout the week. 1 (Monday); 2 (Tuesday); 3 (Wednesday); 4 (Thursday); 5 (Friday); 6 (Saturday); 7 (Sunday). Values are means (standard error); *n* = 35 for AG (active group); *n* = 28 for NAG (non-active group); *n* = 31 for adults without ID. *** Significant difference (*p* ≤ 0.05) between AG vs. NAG.

The analysis using linear mixed-effects models did not show a significant interaction between factor time and group for MVPA (time-by-group: *F* = 0.962, *p* = 0.484). As shown in Figure 3, the AG spent significantly more time performing MVPA than the NAG on Monday, Wednesday, and Friday, corresponding to the days of the PA sessions. No significant differences within group of adults without ID were observed.

**Figure 4:**
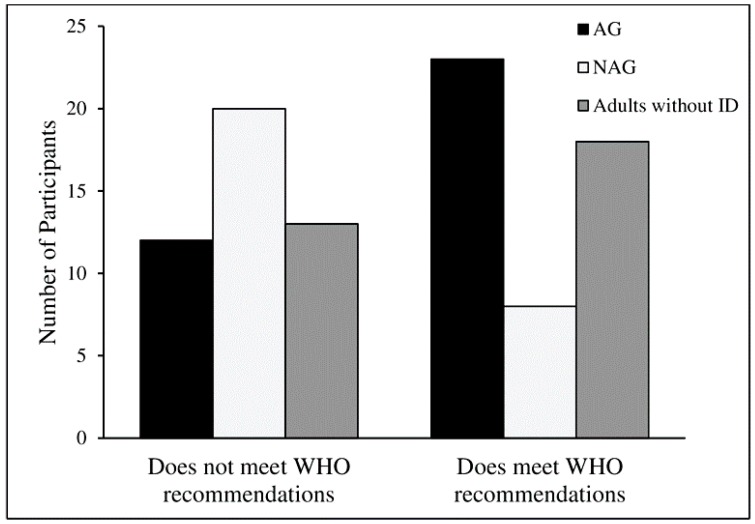
Total number of participants meeting or not meeting the World Health Organization (WHO) global recommendations on PA for health (*X*^2^ = 9.25; *p* = 0.010).

Overall, 49 participants (52.13%) met the WHO global recommendations on PA for health. When divided by groups, 23 participants (65.71%) from the AG, 8 participants (28.57%) from the NAG, and 18 participants (58.06 %) from the group of adults without ID met the WHO recommendations on PA for health (*X*^2^ = 9.25; *p* = 0.010) (Figure 4).

## 4. Discussion

This study provides a better understanding of PA levels and ST in groups of active and non-active adults with ID and a group of adults without ID. The main finding is that participants from the AG engaged in more MVPA than the participants from the NAG only during the days in which they took part in the PA sessions, but the levels of LPA and ST were similar. However, no difference in MVPA was observed between the AG with ID and adults without ID. The adults without ID performed higher amounts of LPA and less ST than both groups of adults with ID.

Females from the non-ID group performed higher amounts of total PA than females from the NAG. The lower total PA in the females from the NAG was a reflection of the low intensity tasks done at the centres and the lack of participation in PA programmes. Males from the AG also performed more MPA and MVPA than males and females from the NAG, which possibly also reflected the nature of their work at the centres and the participation in a PA program.

The groups with ID (active and/or inactive), spent most of their waking time being sedentary, and the ST was similar for both groups, independently of being active or not. This is in line with other studies that have also shown that time spent in sedentary behaviours is higher in adults with ID [19,26,33]. When compared with the group of adults without ID, both groups of adults with ID, regardless of gender and age, were more sedentary and performed less LPA, which concurs with the results of other studies [34,35,36]. In terms of number of bouts in sedentary behaviors a day, it was also not surprising that it was greater in the groups with ID participants, regardless of gender and age, when comparing to the non-ID group. There could be a few contributing factors to this, the ID participants have similar scheduled breaks like tea time and lunch time as dictated by the centres. Also, adults with ID do not have the same opportunities to go to sports facilities after their working day.

In different studies, the percentage of participants with ID achieving WHO recommendations of MVPA ranged from 6% to ~13% [17,19,34]. These values are lower than those obtained in the current study (~49%). The higher percentage of persons with ID meeting WHO recommendations in this study are due to the fact that 23 participants of the AG (~66%) were achieving the recommendations. On the other hand, the number of participants achieving WHO recommendations from the NAG was well below (8 participants; ~29%) the AG. In addition, the number of participants from the groups of adults with and without ID that achieved WHO recommendations on PA for health were higher than the results presented by the Eurobarometer survey on sport and physical activity, where 8% and 33% of European Union citizens were shown to exercise regularly or with some regularity [37].

In concordance with previous studies [19,38,39], this study also showed that participants with ID spent most of their waking time being sedentary (~615 min·day^−1^ for both groups). Moreover, both groups were more sedentary than the adults without ID (~514 min·day^−1^). The average values of LPA (AG = ~117 min·day^−1^ and NAG = ~136 min·day^−1^) performed by participants in our study was similar to values reported by Phillips et al. [39] but higher than the values reported by Melville et al. [38]. Nevertheless, the levels of LPA of the ID participants were lower than the values of the adults without ID.

In terms of MPA and VPA, the average values for MPA (AG = ~37 min·day^−1^ and NAG = ~25 min·day^−1^) were similar to values reported by Phillips et al. [39] whereas the average values for VPA (AG = 1.24 min·day^−1^ and NAG = ~0.67 min·day^−1^) were lower than those reported by the same authors.

Overall, participants from this study engaged in ~33 min of MVPA per day in a week. When dividing the sample by groups, the AG averaged ~39 min of MVPA and the NAG averaged ~26 min of MVPA per day. These values are higher than the results presented by Frey [40] and Melville et al. [38]. However, the values are similar to those achieved by our group of adults without ID and lower than the time spent in MVPA by the active people without ID in the study by Frey [40].

It is important to acknowledge that there are different cut-off points used in these studies. Phillips et al. [39] used the same cut-off points as in the present study but with different epochs (5-s epochs), which may be more sensitive in the temporal spectrum, compared with the longer epochs used in our study. This fact could have led to underestimating the accrued MVPA of the participants in our study. Regarding the study by Melville et al. [38], these authors have used different cut-off points for ST (≤499 counts·min^−1^) and MVPA (>1952 counts·min^−1^) and different epochs (15-s epochs) which may have led them to obtain higher values of ST and MVPA in comparison with our study.

In our study, participants from the AG performed higher values of MVPA, but lower values for LPA and no difference in ST when compared with the NAG. As both groups of adults with ID performed the same tasks at their occupational day centres (like sewing rugs, arts and crafts, weaving baskets, computer work, watching TV or videos, cleaning, and gardening), we could explain that the AG substituted part of their LPA for MVPA due to their participation in the exercise programmes. Also, the shift from LPA to MVPA in the AG made their MVPA levels similar to those of the group of adults without ID, but without affecting or changing their ST or LPA.

Our study showed that during the period of time that the accelerometers were used by the AG, the exercise programmes neither increased their LPA nor decreased their time spent as sedentary, and that the amount of ST that adults with ID accumulate is concerning, particularly if sedentary activities were pursued during the accelerometer’s non-wearing time.

The good news is that research shows that with the first increase in PA beyond the baseline activity, reduction in mortality begins to accumulate [41]. This demonstrates that even small increases in activity could provide substantial health benefits. Furthermore, a meta-analysis [42] found that 60–75 min per day of moderate intensity PA seems to eliminate the increased risk of death associated with high sitting time. However, this meta-analysis also found that watching TV for more than 3 h a day regardless of PA (except in those in the most active quartile) has an increased mortality (Hazard ratio = 1.16, 95%; Confidence interval = 1.05–1.28). These results inform us that health promotion programmes for adults with ID should specifically focus not only on increasing MVPA but on decreasing sedentary activity also, particularly regarding TV watching time. We hypothesize that the accumulation of bouts of exercise lower than 10 minutes may help to improve the health of adults with ID and will be less restricting compared with the general population recommendation [43,44]. This hypothesis needs to be rigorously tested, as exercise needs to be tailored for adults with ID to see what kind of exercise will motivate them the most, maintain their interest, and promote long term sustainability.

We propose that one of the short term goals is to emphasize the importance of decreasing sitting time and begin light PA intensity (1.5–3 METs) and build up intensity to implement MVPA. The ideal PA program should achieve at least 60–75 min/day of moderate intensity PA with less than 3 h per day of TV watching time.

The participation of adults with ID in PA programmes is very low, and this fact may be related to the lack of health promotion culture within health care providers, lack of appreciation of the benefits of PA, and lack of support from their carers [22,45,46]. Generally, research studies in adults with ID have been determining the amount of PA in this population whilst leaving aside the assessment of the real opportunities that these persons have to be involved in PA programmes. The unique characteristics of people with ID and the challenges that they face in terms of participation in structured PA programmes must be assessed. These results must be taken into account in order to develop specific strategies to improve their health.

It is important to acknowledge the limitations of this study, for instance, the PA levels may have been underestimated as the ActiGraph accelerometers were placed on the hip level which meant that movements involving upper limbs were not measured. This, however, is not too concerning, as those activities done with mainly just upper limb, such as reading, playing board games, and computer activity, are considered to be sedentary activities [47]. Furthermore, the accelerometers were not worn during swimming or bathing activities. In this study, the applied cut-off points to estimate PA levels and ST were not established for adults with ID, which could have led to results that may not have been an exact reflection of the PA levels and/or sedentarism of the participants. Given the fact that in the current study people with and without ID of similar ages were compared, and taking into account that people with ID suffer from an early aging process starting around age 40–50 [23,24,25], future studies should also compare PA levels and ST of adults with ID older than 40–45 versus adults without ID older than 65 years old. Finally, interpretation of these results should be treated with caution, owing to the small sample of adults with ID included in this study.

## 5. Conclusions

When assessing ST and PA levels in active and non-active ID participants, it was observed that participants presented large amounts of sedentary behaviours in both groups. The participants of the AG, despite participating in PA programmes and mostly meeting the WHO guidelines on PA for health, did not demonstrate less ST. Also, as showed in our study, the PA programmes where the AG were involved allowed them to achieve PA levels similar to those from the non-ID group, but the amount of time spent in ST and the levels of LPA were still lower than those from the adults without ID.

An increase in PA will not only help to reduce secondary complication (obesity, hypertension, poor nutritional habits, and psychological problems), it also has an important role in reducing chronic diseases, which will have a significant impact in the population with ID. We believe that with the support of the public and local administrations, including well-designed and structured PA programmes into adults with ID’s workdays, as well as the incorporation of breaks to reduce ST, a system that greatly helps to increase daily PA levels of people with ID can be developed.

## Figures and Tables

**Table 1:** Distribution of the participants with intellectual disabilities (ID) over the groups.

Level of ID	AG (*n* = 37)	NAG (*n* = 29)	*p*-Value
Participants with mild ID level	11	7	0.613
Participants with moderate ID level	26	22	

Abbreviations: ID (intellectual disability); AG (active group with ID); NAG (non-active group with ID). Between-group differences was based on a chi-square test.

**Table 2:** Participants’ characteristics, anthropometry indices and PA data.

Variables	Adults without ID (*n* = 31)	AG (*n* = 37)	NAG (*n* = 29)	*F*	*p*-Value
**Characteristics ^‡^**					
Age (years)	43 (11)	41 (11)	46 (12)	1.657	0.196
Gender (male/female)	14/17	22/15	17/12		
**Anthropometry ^‡^**					
Height (cm) *	167.90 (7.79)	160.43 (10.87)	162.03 (9.30)	4.241	**0.017**
Weight (kg)	73.64 (12.04)	70.07 (13.55)	74.24 (12.52)	1.061	0.350
BMI (kg/m^2^)	26.08 (3.71)	27.38 (5.00)	28.56 (6.35)	1.780	0.174
**PA data ^†; ‡‡^**					
Total PA (counts·min^−1^) **; ***	316.86 (78.71)	306.86 (85.71)	236.54 (107.90)	6.701	**0.002**
ST (min·day^−1^) *; **	513.71 (81.88)	614.98 (106.77)	615.04 (80.57)	44.225	**<0.001**
LPA (min·day^−1^) *; **	252.39 (69.78)	117.37 (39.57)	136.69 (49.92)	57.346	**<0.001**
MPA (min·day^−1^) ***	33.92 (17.35)	37.48 (26.29)	25.26 (19.87)	2.759	0.069
VPA (min·day^−1^) *; ***	0.69 (1.08)	1.24 (0.99)	0.67 (0.45)	3.240	**0.044**
MVPA (min·day^−1^) ***	34.61 (17.08)	38.72 (26.64)	25.95 (20.58)	2.929	0.059
Sedentary bouts >1 min per day *; **	99.76 (23.64)	123.34 (20.13)	124.69 (20.79)	6.636	**0.002**
Sedentary breaks/sedentary hour	11.26 (2.16)	11.64 (1.80)	12.37 (2.15)	1.608	0.206
Accelerometer wearing time (min·day^−1^)	800.72 (76.14)	771.08 (111.52)	777.68 (73.10)	0.886	0.416

Note: values are means (standard deviation). Abbreviations: ID (intellectual disability); AG (active group with ID); NAG (non-active group with ID); BMI (body mass index); PA (physical activity); ST (sedentary time); LPA (light physical activity); MPA (moderate physical activity); VPA (vigorous physical activity); MVPA (moderate to vigorous physical activity). ^†^ *n* = 35 for AG; *n* = 28 for NAG. ^‡^ Between-group differences based on ANOVA analysis. ^‡‡^ Between-group differences based on one-way analysis of covariance (ANCOVA) analysis adjusted for accelerometer wearing time. Statistically significant trends are showed in bold (*p* ≤ 0.05). * Significant difference (*p* ≤ 0.05) between adults without ID vs. AG. ** Significant difference (*p* ≤ 0.05) between adults without ID vs. NAG. *** Significant difference (*p* ≤ 0.05) between AG vs. NAG.

**Table 3:** Patterns of sedentary time and physical activity levels by group and sex.

Variables	Adults Without ID		AG		NAG		*F*	*p*-Value
	Males (*n* = 14)	Females (*n* = 17)	Males (*n* = 21)	Females (*n* = 14)	Males (*n* = 17)	Females (*n* = 11)		
Total PA (counts·min^−1^) *; ***; †	325.11 (74.24)	310.06 (83.85)	314.61 (96.18)	295.24 (68.85)	245.25 (126.56)	223.09 (74.03)	2.814	**0.021**
ST (min·day^−1^) ****	505.29 (57.93)	520.65 (98.65)	620.14 (100.49)	607.23 (119.05)	600.26 (86.47)	637.87 (67.96)	17.951	**<0.001**
LPA (min·day^−1^) ****	234.14 (67.41)	267.41 (70.05)	121.51 (47.83)	111.14 (22.48)	140.64 (58.29)	130.57 (35.01)	23.251	**<0.001**
MPA (min·day^−1^) †	33.93 (15.33)	33.92 (19.33)	43.49 (30.48)	28.47 (15.24)	24.72 (23.22)	26.10 (14.22)	1.746	0.133
VPA (min·day^−1^) **; ††; †††	1.17 (1.83)	0.29 (0.69)	1.40 (1.12)	1.00 (0.74)	0.66 (.49)	0.69 (0.39)	2.824	**0.021**
MVPA (min·day^−1^) †	35.10 (14.31)	34.21 (19.51)	44.89 (30.79)	29.47 (15.62)	25.46 (24.07)	26.70 (14.72)	1.857	0.110
Sedentary bouts >1 min per day ****	98.47 (22.99)	100.81 (25.87)	124.16 (19.56)	122.15 (21.58)	125.60 (24.30)	123.28 (17.81)	2.593	**0.031**
Sedentary breaks/sedentary hour	11.86 (2.35)	10.77 (1.93)	11.41 (1.96)	11.97 (1.57)	12.33 (2.39)	12.43 (4.21)	1.038	0.400
Accelerometer wearing time (min·day^−1^)	794.55 (69.65)	802.27 (76.39)	786.56 (110.17)	747.85 (116.77)	766.77 (73.57)	795.16 (72.24)	1.183	0.324

Note: values are means (standard deviation). Abbreviations: ID (intellectual disability); AG (active group with ID); NAG (non-active group with ID); PA (physical activity); ST (sedentary time); LPA (light physical activity); MPA (moderate physical activity); VPA (vigorous physical activity); MVPA (moderate to vigorous physical activity). Between-group differences based on ANCOVA analysis adjusted for accelerometer wearing time. Statistically significant trends are showed in bold (*p* ≤ 0.05). * Significant difference (*p* ≤ 0.05) between females without ID vs. females from the NAG. ** Significant difference (*p* ≤ 0.05) between males without ID vs. females from the NAG. *** Significant difference (*p* ≤ 0.05) between males without ID vs. males and females from the NAG. **** Significant difference (*p* ≤ 0.05) between males and females without ID vs. males and females from the AG and NAG. † Significant difference (*p* ≤ 0.05) between males from the AG vs. males and females from the NAG. †† Significant difference (*p* ≤ 0.05) between males and females from the AG vs. females from the NAG. ††† Significant difference (*p* ≤ 0.05) between males from the AG vs. males from the NAG.

**Table 4:** Patterns of sedentary time and physical activity levels by group and age.

Variables	Adults Without ID		AG		NAG		*F*	*p*-Value
	<40 years (*n* = 15)	≥40 years (*n* = 16)	<40 years (*n* = 18)	≥40 years (*n* = 17)	<40 years (*n* = 16)	≥40 years (*n* = 12)		
Total PA (counts·min^−1^) *; **; †	335.53 (89.09)	299.35 (65.63)	329.79 (97.13)	282.57 (66.07)	242.74 (96.43)	228.28 (125.56)	3.546	**0.006**
ST (min·day^−1^) ***	497.76 (79.61)	528.67 (83.67)	601.01 (106.98)	631.54 (87.09)	617.61 (80.17)	611.61 (84.54)	18.731	**<0.001**
LPA (min·day^−1^) ***	267.74 (60.69)	237.99 (76.45)	110.74 (39.62)	124.38 (39.45)	125.27 (45.35)	151.91 (53.58)	23.995	**<0.001**
MPA (min·day^−1^)	36.72 (20.64)	31.34 (13.47)	40.69 (32.25)	34.08 (18.44)	27.93 (22.34)	21.69 (16.27)	2.115	0.071
VPA (min·day^−1^) †; ††	0.99 (1.76)	0.36 (0.74)	1.48 (0.90)	0.99 (1.01)	0.72 (0.45)	0.62 (0.46)	2.467	**0.039**
MVPA (min·day^−1^)	37.71 (20.79)	31.71 (12.72)	42.17 (32.41)	35.07 (19.10)	28.80 (23.19)	22.15 (16.71)	2.219	0.059
Sedentary bouts >1 min per day ***	94.04 (18.23)	104.80 (26.60)	115.04 (18.69)	132.67 (17.87)	120.69 (14.96)	128.15 (24.79)	4.240	**0.002**
Sedentary breaks/sedentary hour	11.17 (1.77)	11.35 (2.53)	11.79 (2.12)	11.47 (1.41)	12.09 (2.76)	12.59 (3.33)	1.467	0.209
Accelerometer wearing time (min·day^−1^)	803.22 (84.71)	798.37 (69.89)	753.53 (106.18)	791.01 (112.80)	771.69 (81.24)	785.68 (63.21)	1.357	0.239

Note: values are means (standard deviation). Abbreviations: ID (intellectual disability); AG (active group with ID); NAG (non-active group with ID); PA (physical activity); ST (sedentary time); LPA (light physical activity); MPA (moderate physical activity); VPA (vigorous physical activity); MVPA (moderate to vigorous physical activity). Between-group differences based on ANCOVA analysis adjusted for accelerometer wearing time. Statistically significant trends are showed in bold (*p* ≤ 0.05). * Significant difference (*p* ≤ 0.05) between participants ≥40 years from the group of adults without ID vs. participants ≥40 years from the NAG. ** Significant difference (*p* ≤ 0.05) between participants <40 years from the group of adults without ID vs. participants <40 years and ≥40 years from the NAG. *** Significant difference (*p* ≤ 0.05) between participants <40 years and ≥40 years from the group of adults without ID vs. participants <40 years and ≥40 years from the AG and NAG. † Significant difference (*p* ≤ 0.05) between participants <40 years from the AG vs. participants <40 years and ≥40 years from the NAG. †† Significant difference (*p* ≤ 0.05) between participants <40 years from the AG vs. participants ≥40 years from group of adults without ID.
